# Editorial: Molecular mechanisms regulating phenotypic heterogeneity in human inflammatory diseases

**DOI:** 10.3389/fimmu.2023.1214255

**Published:** 2023-05-17

**Authors:** Theodore S. Kapellos, Martijn C. Nawijn

**Affiliations:** ^1^ Comprehensive Pneumology Center (CPC), Institute of Lung Health and Immunity (LHI), Helmholtz Munich, Member of the German Center for Lung Research (DZL), Neuherberg, Germany; ^2^ Department of Pathology and Medical Biology, University of Groningen, University Medical Center Groningen, Groningen, Netherlands; ^3^ GRIAC Research Institute, University Medical Center Groningen, Groningen, Netherlands

**Keywords:** myeloid cells, inflammation, single-cell ‘omics, human disease, granulocytes

Myeloid cells (monocytes, macrophages, dendritic cells, mast cells and granulocytes) are bone marrow-derived cells of the innate immune system and function as the first responders during acute infectious and sterile inflammatory insults. However, when their responses are not tightly regulated, the activity of these innate immune cells can have detrimental effects on tissue integrity and trigger chronic inflammatory conditions.

The advent of omics and spatial technologies complemented by the work carried out within international consortia, such as the Human Cell Atlas (1) and the Immunological Genome project (2) have sparked renewed appreciation for the diversity of myeloid cells, both in the circulation and as tissue-resident cells in all organs (3). Single-cell omics datasets now provide a new perspective on myeloid cells, including their developmental trajectories in the bone marrow, their cell identities defined by transcriptional and epigenetic features and the origins of tissue-resident myeloid populations in homeostasis (4).

In addition to the description of myeloid cells in health, several studies have focused on the role of myeloid cells in human inflammatory diseases (4, 5). Among others, specific disease-associated myeloid populations have been described in the lung and the brain, in peripheral blood and in conditions, such as pulmonary fibrosis (6), COVID-19 (7), cancer (8–11) and Alzheimer’s disease (12, 13). What is, however, still missing is a better understanding of the implications of these disease-associated myeloid cell subsets to the disease process, including progression and remission or treatment response and the identification and validation of biomarkers that may be utilized for diagnosis or prognosis of clinical outcome. A comprehensive analysis of myeloid cell diversity is critical for novel drug discovery, as such data will not only allow for the identification of molecular pathways that are altered in disease, but also for the establishment of *ex vivo* models to validate both their causative role to disease and the effects of the disease microenvironment on their biological functions in perturbation experiments. Moreover, additional single-cell transcriptomics, epigenetics and proteomics datasets on myeloid cells can now be fully integrated with single-cell atlases (14) in order to increase the resolution of these references, expand them with additional modalities and improve their statistical power for comparisons between relevant clinical groups or even across diseases, as exemplified by the fibrosis-associated monocyte-derived macrophage subset described in the Human Lung Cell Atlas which was observed to be present in lung tissue from patients with interstitial lung disease and long COVID-19 (15).

To this end, the goal of this Research Topic was to highlight the phenotypic heterogeneity of tissue-resident and peripheral myeloid populations and characterize the pathways that a) are dysregulated in chronic human inflammatory diseases and b) may have predictive value in distinguishing disease phenotypes. Our collection includes 4 primary research articles that touch on the heterogeneity and functionalities of myeloid cells in severe burn, chronic obstructive pulmonary disease (COPD), sepsis and *Mycobacterium tuberculosis* infection.

Circulating neutrophils are an understudied immune population despite their high abundance in the peripheral blood and their involvement in systemic diseases, such as sepsis and severe burns. Huang et al. analyzed the transcriptional landscape of circulating neutrophils from early-stage severe burn patients and discovered distinct subgroups with functional differences. The authors described the upregulated degranulation and metabolic dysregulation and observed time-dependent patterns of activation, suggesting a role for neutrophils in early stages of severe burn. These findings are in line with Velásquez et al. who followed up the frequencies and gene expression profiles of blood CD15^+^ cells to distinguish systemic inflammatory response syndrome from sepsis patients in the intensive care unit. The presence of immature neutrophil populations (promyelocytes, myelocytes) defined the clinical picture in sepsis patients, a finding that was reflected by the elevation of azurophilic, specific and gelatinase-containing granule gene expression. Together, the data speak for the significance of neutrophil gene signatures as predictive biomarkers in systemic inflammatory diseases.

Lung macrophage populations play a pivotal role in mounting immune responses against invading pathogens and irritants, such as cigarette smoke. In our collection, Magoulopoulou et al. and Baßler et al. contributed two exciting stories about the transcriptional remodeling of these myeloid cells in infection and COPD. Using spatial transcriptomics technologies and a 33-gene set, Magoulopoulou et al. demonstrated that inflammatory macrophages are located at subcellular distance to *Mycobacterium tuberculosis* bacilli, reminiscent of infected populations, whereas non-infected cells were enriched in antigen presentation and T cell-related transcripts at later time points. The results reveal differences in spatial distribution between infected macrophage populations and T cells, as well as time-dependent induction of antigen presentation and adaptive immunity programs in tuberculosis. Lastly, Bassler et al. assessed the population structure of alveolar macrophages in COPD, a progressive obstructive and inflammatory condition of the small airways, with single-cell transcriptomics. They showed that macrophages from COPD patients decrease antigen presentation capacity and chemotaxis, accumulate cholesteryl esters and undergo mitochondrial dysfunction, reminiscent of impaired immune activation. Cell-cell communication analysis predicted that TGF-β is a major upstream regulator of transcriptional changes. Both articles highlight the high degree of macrophage plasticity in infection and chronic inflammatory diseases and suggest that further work is required to better understand the relationship between their functions and the exact localization in their microenvironment.

In conclusion, our Research Topic aimed to shed light on the transcriptomic diversity of myeloid populations and provide evidence of gene expression profiles that may serve as biomarkers for human inflammatory diseases. Indeed, the included work studied the functions of blood neutrophil subgroups and their gene signatures in severe burn and sepsis, while making a case about the relevance of tissue localization in shaping macrophage responses in tuberculosis and the complex transcriptional remodeling in macrophages from human restrictive lung disease.

## Author contributions

TSK wrote the editorial. All authors contributed to the article and approved the submitted version.
